# Free Volatile Compounds of *Veronica austriaca* ssp. *jacquinii* (Baumg.) Eb. Fisch. and Their Biological Activity

**DOI:** 10.3390/plants10112529

**Published:** 2021-11-20

**Authors:** Marija Nazlić, Željana Fredotović, Elma Vuko, Nenad Vuletić, Ivica Ljubenkov, Dario Kremer, Renata Jurišić Grubešić, Edith Stabentheiner, Marko Randić, Valerija Dunkić

**Affiliations:** 1 Faculty of Science, University of Split, Ruđera Boškovića 33, HR-21000 Split, Croatia; mnazlic@pmfst.hr (M.N.); zfredotov@pmfst.hr (Ž.F.); elma@pmfst.hr (E.V.); nenov@pmfst.hr (N.V.); iljubenk@pmfst.hr (I.L.); 2 Faculty of Pharmacy and Biochemistry, University of Zagreb, A. Kovačića 1, HR-10000 Zagreb, Croatia; dkremer@pharma.hr; 3 Faculty of Medicine, University of Rijeka, Braće Branchetta 20, HR-51000 Rijeka, Croatia; renatajg@medri.uniri.hr; 4 Institute of Biology, Karl-Franzens University, Schubertstrasse 51, A-8010 Graz, Austria; edith.stabentheiner@uni-graz.at; 5 Biology Public Institution “Priroda”, Grivica 4, HR-51000 Rijeka, Croatia; marko.randic@ju-priroda.hr

**Keywords:** *Veronica*, volatile compounds, GC-MS, antioxidant activity, DPPH, ORAC, essential oil, hydrosol, antiproliferative activity, PCA

## Abstract

The composition of free volatile compounds of essential oils (EO) and hydrosols (Hy) from four different localities of the species *Veronica austriaca* ssp. *jacquinii* (Baumg.) Eb. Fisch. were analyzed by gas chromatography coupled with mass spectrometry. In the EOs, the most abundant compounds identified were hexahydrofarnesyl acetone (23.34–52.56%), hexadecanoic acid (palmitic acid, 26.71–58.91%) and octadecanol acetate (0–6.24%). The hydrosols were characterized by high abundance of methyl eugenol (23.35–57.93%), *trans*-*p*-mentha-1(7),8-dien-2-ol (5.24–7.69%) and thymol (3.48–9.45%). Glandular trichomes were analyzed using SEM (Scanning Electron Microscopy), as they are the sites of synthesis of free volatile compounds. We have detected glandular trichomes, consisting of a one stalk cell and two elliptically shaped head cells, and non-glandular (unbranched, bi-cellular to multicellular) trichomes on stems, leaves and the sepals. Data for volatile compounds from EOs and hydrosols were analyzed using Principal Component Analyses (PCA) to demonstrate variations in the composition of the volatile compounds identified. Isolated samples of EO and hydrosols were analyzed for their antioxidant activity using two methods, DPPH (2,2-diphenyl-1-picrylhydrazyl) and ORAC (Oxygen Radical Absorbance Capacity). The essential oils showed higher antioxidant activity than the hydrosols in ORAC method, but lower activity by the DPPH method. The isolates were also tested for their antiproliferative activity on different types of cancer cells and also on two lines of healthy cells, and the results showed that the extracts were not toxic to the cell lines tested. Total polyphenols, total tannins, total flavonoids and total phenolic acids were also analyzed and determined spectrophotometrically. The free volatile compounds of *Veronica austriaca* ssp. *jacquinii* can be considered as a safe natural product.

## 1. Introduction

Genus *Veronica* L. is divided into 13 subgenera according to the latest classifications and belongs to the Plantaginaceae family (formerly belonged to Scrophulariaceae) [1]. The large number of species (about 500) is an indication of the high ecological adaptability of this genus, in which species grow in wet and dry habitats, by the sea and in the mountains [2,3], but they mostly grow in regions with a Mediterranean precipitation regime [4]. In Croatia there are about 40 species of *Veronica* [5].

**Figure 1:**
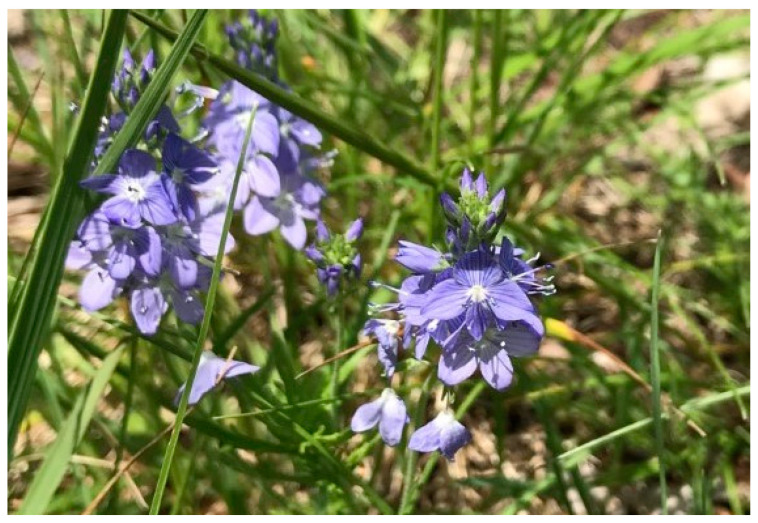
*Veronica austriaca* ssp. *jacquinii* in its natural habitat (Lika, Croatia).

Species *Veronica austriaca* ssp. *jacquinii* (Baumg.) Eb. Fisch. (Figure 1) is a perennial herbaceous plant with an oblique, cylindrical rhizome. There are numerous stems, or only one, upright, tall (10) 30–70 cm, covered with gray hairs. Leaves are round or broadly lanceolate, pinnately lobed, pinnately or double-pinnately divided. The lobes are narrow, linear or linearly lanceolate, entire or serrated, more or less in-rolled, hairy or rarely glabrous. The leaves of the sterile part of the shoot at the top of the stem are almost whole. The flowers are arranged in 2–4 lateral, opposite racemes. The seeds are flattened, almost round [6].

The literature search revealed that the most studied specialized metabolites of *V. austriaca* ssp. *jacquinii* and the genus *Veronica* in general include iridoid glucosides, phenylethanoids and flavonoid glycosides [7], so this is the first study on the chemical composition of essential oils (EO) and hydrosols from this species. Živković et al. studied phenolic compounds, antioxidative and antineurodegenerative effect of *V. austriaca* ssp. *jacquinii* and their results showed that this species has significant antioxidant and antineurodegenerative activity [8]. Many other *Veronica* species and the biological activity of their specialized metabolites, especially phenolic compounds, were investigated and the experiments showed that they have antioxidant [8,9,10,11,12], antimicrobial [7,11,13,14], cytotoxic and antitumor activities [13]. All these studies were performed with some kind of phenolic extract prepared using different solvents (methanol, ethanol, acetone or water). Hydrosols, that are the subject to this research, are condensed water vapors containing dissolved molecules of essential oils and more water-soluble (polar) volatile compounds [15]. Due to the difference in solubility of volatile compounds in water, the entire composition and hence biological activity of hydrosols differ from that of essential oils. Hydrosols are often discarded after essential oil extraction, but studies show that these waste products are rich in biologically active substances [15,16,17] so their potential use should be further researched. Essential oils are a very complex mixture of compounds, mainly monoterpenes and sesquiterpenes, and currently more than 3000 essential oils are investigated for their composition but not all of them have shown significant biological activity, only one tenth [18], so it is very important to continue investigating plant volatiles. Due to the natural richness in phenolic compounds and iridoids, species from the genus *Veronica* are widely used in traditional medicine in treating various diseases, influenza, respiratory diseases, cancer and as diuretic [14,19] and this is probably the reason why the interest in the chemistry and biological activity of the specialized metabolites of this genus began at the beginning of the last century. In the traditional medicine of the Balkan people, the aboveground parts of the species *V. officinalis* are used to treat liver, spleen, kidney, and bladder diseases, for the treatment of snake bites, for wound healing, skin damage, eczema, and ulcers [9,14,20]. Since the biological activity of the volatile compounds of the genus *Veronica* (apart from our earlier research on *V. saturejoides* ssp. *saturejoides*) has not been investigated to date, our team decided to investigate the biological activity of the extracted volatile compounds as well. Therefore, the aim of this study was to investigate the volatile compounds of this species especially with regard to comparison of the volatile components of essential oil and of water residues (hydrosols), as well as discussing differences and similarities in the composition, taking into account the different locations where the plant material was collected. To our knowledge, this is the first report on the composition of the essential oil and hydrosols and their biological activity, as well as on the micromorphology of the trichomes of *V. austriaca* L. ssp. *jacquinii*.

## 2. Results and Discussion

### 2.1. Volatile Compounds from Essential Oils and Hydrosols

The isolation and identification of the four samples of essential oils and four related hydrosols were determined by GC and GC-MS; the results are calculated as relative amounts of the compounds expressed in percentage and are reported in the Table 1 and Table 2. The compounds are listed in the order of their elution from the column. The yields of EOs from four locations were 0.38%, 0.47%, 0.51% and 0.64%, respectively. The objective was to determine the similarities and differences in the volatile components depending on the population. In all EO samples, more than 90% of the total oil was identified, with hexahydrofarnesyl acetone (23.34%–52.56%) and hexadecanoic acid (26.71%–58.91%) being the most abundant (Figure S1). These components were also identified in all hydrosol samples, but in much lower percentage ranging from 0.48% to 7.70% when both components are observed. Apart from these two components, *E*-caryophyllene and (*Z)*-methyl isoeugenol are present in all four EO samples and in all four hydrosol samples. The major components in the hydrosols are methyl eugenol (23.35%–57.93%), *trans*-*p*-mentha-1(7),8-dien-2-ol (5.24%–7.69%) and thymol (3.48%–9.45%) (Table 1 and Table 2).

**Figure 2:**
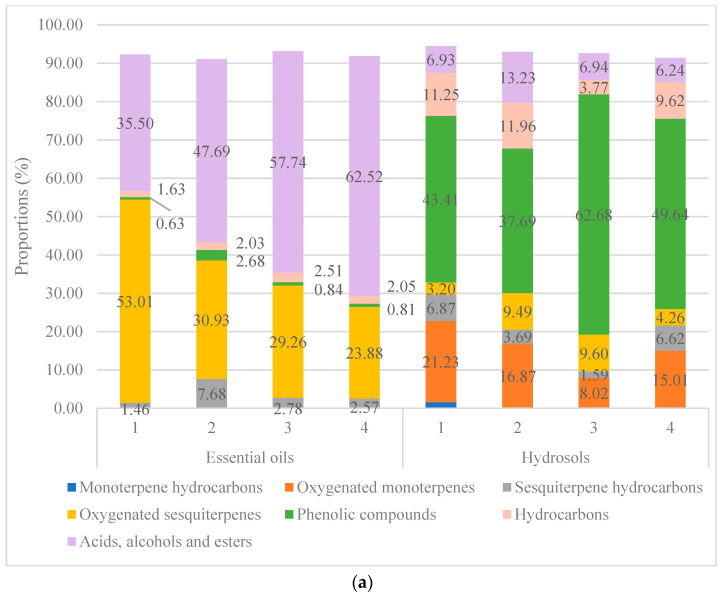

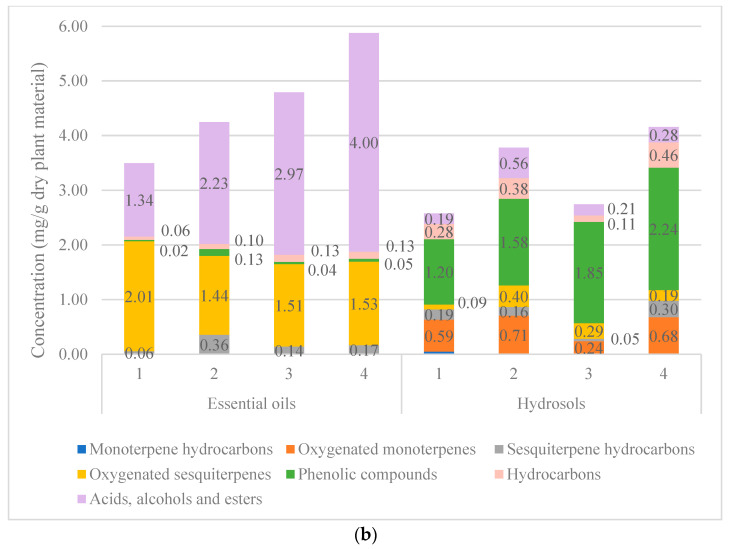
Volatile compounds distribution by categories for all samples of essential oils and hydrosols from four locations; (**a**) based on the relative peak area identification (data from Table 1 and Table 2); (**b**) based on the concentration of volatile components in dry plant material (data from Tables S1 and S2).

In the Figure 2a composition is presented based on the Table 1 and Table 2 (according to %—relative peak area). It can be seen from the Figure 2a. that the composition of all the EO samples is characterized by high percentage of “Oxygenated sesquiterpenes” and “Acids, alcohols and esters categories”. This is in agreement with our previous results for the EOs of *V. saturejoides* [12]. According to a literature review, GC-MS studies have been performed on only a few *Veronica* species. Ertas et al. found that the most abundant constituent of the essential oil from *Veronica thymoides* subsp. *pseudocinerea* is hexatriacontene (21%) which belongs to the hydrocarbon compound group [9]. In our previous research in the oil of *V. spicata* L., the most abundant compound was the diterpene phytol [14]. In another research of the essential oil composition of *Veronica linariifolia* Pall. ex Link the major constituents were cyclohexene (25.83%), *β*-pinene (11.61%), 1S-*α*-pinene (10.65%), *β*-phellandrene (10.49%), *β*-myrcene (10.42%), and germacrene D (4.99%) (monoterpene and sesquiterpene hydrocarbons) [21]. Çelik et al. studied the essential oils extracted from *Veronica* sp., and found that the main components were mainly linalool (4.18%) and carvacrol (7.28%) [22]. Valyova et al. studied extracts of the Bulgarian species *Veronica officinalis* by GC-MS and found the following composition in was found in the ethanol extract of the aboveground parts: terpenes, saturated and unsaturated fatty acids and esters, steroids, p-hydroxyphenylethyl alcohol, maltol and loliolide. In terms of content, *β*-sitosterol was the most abundant. In their study, they also identified terpinen-4-ol, neophytadiene, hexahydrofarnesyl acetone, vitamin E, phytol and squalene for the first time in the genus *Veronica* [23]. As in the above-mentioned research, we have also found hexahydrofarnesyl acetone in both *V. saturejoides* and *V. jacquinii*, and it was in both species the main EO compound. In addition to this compound, hexadecanoic (palmitic) acid was also found in high percentage in all the EO samples examined. All four samples of the hydrosols of *V. austriaca* ssp. *jacquinii* have similar composition with methyl eugenol as the most abundant compound (Table 2, Figure S2). This compound could be responsible for the higher antioxidant activity of the hydrosol compared to the EO activity (measured with the DPPH method). The main difference between the composition of EO and hydrosol (Figure 2) is that non-polar compounds such as fatty acids and oxygenated sesquiterpenes are the main compound categories in EOs, whereas more polar compounds such as phenolic acids and oxygenated monoterpenes are the most abundant in hydrosols. Other specialized metabolites have also been studied for this species. Živković et al. discovered flavonoids derived from flavones—luteolin and isoscutellarein and found that acteoside is the most dominant compound in *V. austriaca* ssp. *jacquinii*. They also detected quercetin derivates in this species [8].

In the Supplementary Materials in the Tables S1 and S2 we have recalculated compositions of volatile compounds from EOs and hydrosols according to the yields from dry plant material. The main categories are presented in the Figure 2b. If we compare data from Figure 2a,b, the main difference is that in the Figure 2b it is well presented that concentration of volatiles in hydrosols is lower than in the EOs but the distribution of the main categories remains the same as they are distributed in the Figure 2a. It can be seen that in the EOs compounds belonging to the “Oxygenated sesquiterpenes” and “Acids, alcohols and esters” are the most abundant (1.44–2.01 and 1.34–4.00 mg/g, respectively) and compounds belonging to the “Phenolic compounds” are the most abundant in the hydrosols (1.20–2.24 mg/g).

### 2.2. Principal Component Analyses

**Figure 3:**
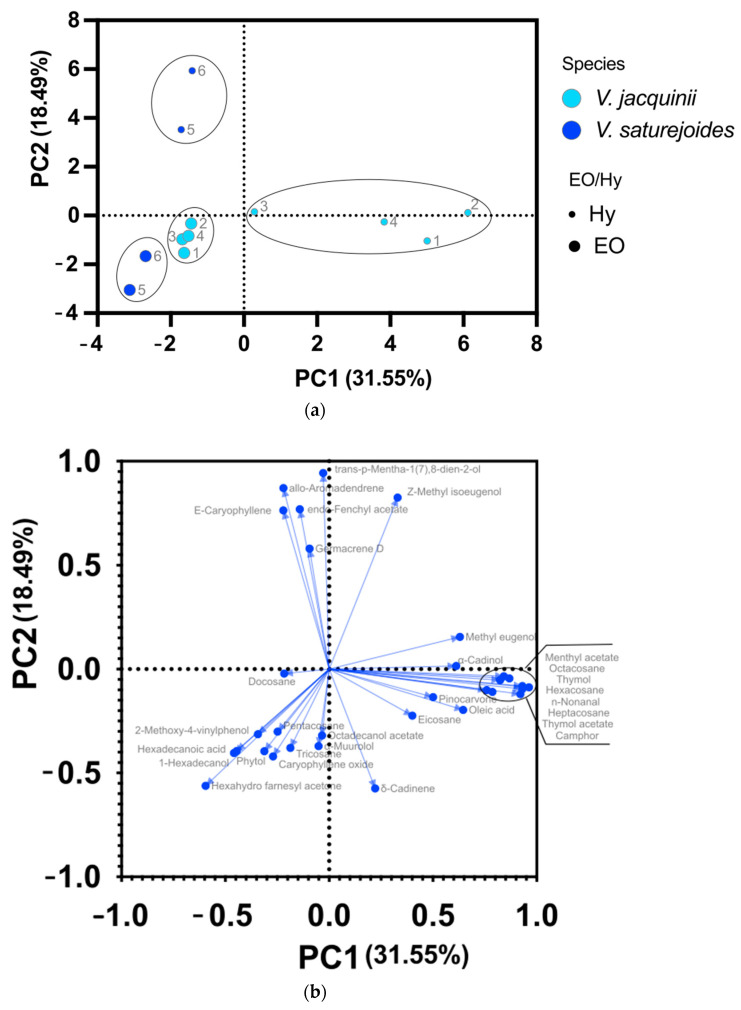
PCA analyses of volatile compound in the amount larger than 2% from essential oils (EO) and hydrosols (Hy) of *V. austriaca* ssp. *jacquinii* from four locations. (**a**) PCA score plot allocating different species into clusters; (**b**) PCA loading plots of volatiles from the first and second principal component.

PCA analyses were performed for volatile compounds from EOs and hydrosols with the amount greater than 2% (Figure 3). In addition to volatiles from the *V. austriaca* ssp. *jacquinii* from this research we also included volatile compounds of *V. saturejoides* from our previous research that is presented in Table 1 in Nazlić et al. [12] to see whether the two species will differentiate from each other and if volatile compounds could be a distinguishing feature for this genus (Figure 3a). PC1 and PC2 for volatile compounds from EOs and hydrosols explained 50.04% of the variance and distinguished *V. saturejoides* from *V.austriaca* ssp. *jacquinii*. In addition, hydrosols were distinguished from the EOs. This was to be expected, as mentioned earlier, the composition of volatile compounds in the EOs is somewhat different than in the hydrosols. The components differentiating EOs from hydrosols are located in the negative region of PC1 and PC2 for both species. The major components that differentiate *V. austriaca* ssp. *jacquinii* from *V. saturejoides* for hydrosols are *trans*-*p*-1(7),8-mentha-dien-2-ol, *allo*-aromadendrene, *Z*-methyl isoeugenol, germacrene D and *E*-caryophyllene (Figure 3b). The main components that differentiate *V. austriaca* ssp. *jacquinii* from *V. saturejoides* for the EOs are 1-hexadecanol, α-muurolol, tricosane, pentacosane, 2-methoxy-4-vinylphenol, octadecanol acetate, *β*-caryophyllene oxide and hexadecanoic acid (Figure 3b). From the Figure 3, it can be seen that the two species are best distinguished on the basis of the volatile components of the hydrosols. To our knowledge, this is the first PCA analysis of volatile compounds for the genus *Veronica*. However, numerous other experiments have been conducted for other species, suggesting that volatile compounds can be used as a discriminating factor between species/cultivars [26,27,28].

### 2.3. Micromorphology of the Trichomes

**Figure 4:**
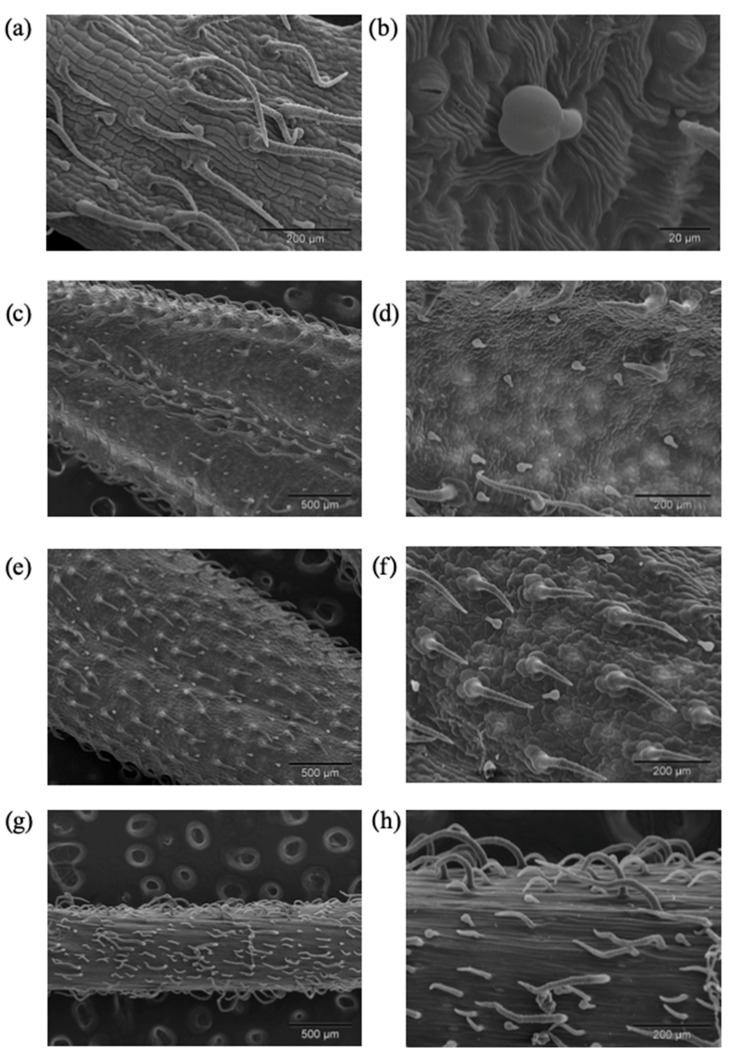
*V. austriaca* ssp. *jacquinii* SEM micrographs showing differences in the distribution of the trichomes on sepal (**a**,**b**), abaxial (**c**,**d**) and adaxial (**e**,**f**) leaf surface, and stem (**g**,**h**).

Glandular trichomes are part of plant organism where compounds that make essential oils are synthesized. Their role in protecting plants from herbivores and pathogens is very important and as pollinator attractors as well [29]. Volatile compounds from EOSs are generally less investigated than some other specialized metabolites so this is also the reason why we chose to investigate the cells in which these compounds are produced. Both non-glandular and one type of glandular trichomes were observed on calyxes, leaves and the stems of *V. austriaca* ssp. *jacquinii*. According to SEM-investigation non-glandular trichomes vary from bi-celled to multicelled (Figure 4a,f,h). They are unbranched, uniseriate and folded at different levels, while the length varied from short to long hairs. Their surface showed a warty appearance due to the presence of cuticular micropapillae (Figure 4a). These type of trichomes could be noted as attenuate hairs [30]. Calyxes, stems and adaxial leaves side were moderately dense covered by non-glandular trichomes (Figure 4a,e,h), while on the abaxial leaves side these trichomes were mainly distributed along the main vein and leaf edge (Figure 4c). This type of non-glandular hairs was described in our previous research on the *V. saturejoides* ssp. *saturejoides* [12]. Existence of non-glandular trichomes on flower parts of *Veronica* species was mentioned by Kurer [31]. Kraehmer and Baur found non-glandular trichomes in *V. persica* Poir. [32]. Attenuate, non-glandular trichomes are commonly known from other plant species [33,34,35].

The glandular trichomes of *V. austriaca* ssp. *jacquinii* belong to capitate type of trichomes and consist of one stalk cell and two elliptically shaped head cells (Figure 4b). These trichomes were not upright but could be described as clinging to the surface. They were observed on calyxes, leaves and the stems. All investigated plant parts were moderately dense covered by capitate trichomes. This type of glandular trichomes was also noted by our team in *V. saturejoides* ssp. *saturejoides* [12]. Kristen and Lockhausen (1985) found the same type of capitate trichomes in *Veronica beccabunga* L. [36]. A glandular, capitate, inclined trichome type with a bicellular head is also known from *Stachys recta* L. ssp. *recta* [37]. Haratym and Weryszko-Chmielewska found the same type of capitate trichomes with two-celled head on the stem and leaves of *Marrubium vulgare* L. (Lamiaceae) [38]. Comparable glandular trichomes, but with only one elliptically shaped head cell, were found on *Satureja thymbra* L., *Thymus capitatus* (L.) Hoffmanns, *Majorana syriaca* (L.) Rafin. [33], *Calamintha menthifolia* Host. [39], *Geranium macrorrhizum* L. and *G. dalmaticum* (Beck) Rech. f. [40]. In general comparison with the previously investigated *V. saturejoides* [12], we can say that the trichomes of *V. austriaca* ssp. *jacquinii* are denser on all parts of the above ground parts (stem, leaves and calyxes).

### 2.4. Phenolic Compounds in Hydrosols

The phenolic compounds in the hydrosols were also investigated by the HPLC method to compare the results with our previous studies on *V. saturejoides*. We did not find any phenolic compounds in any of the samples examined from four sites of *V. austriaca* ssp. *jacquinii*. In our previous research, in one sample of *V. saturejoides* we detected vanillin, cinnamic acid and protocatechuic acid. The hydrosol sample from this location had higher antioxidant activity than the plant sample of the same species, but from different location, which did not contain any phenolic compounds [12]. When compared with the results for *V. austriaca* ssp. *jacquinii*, the hydrosol sample of *V. saturejoides* in which phenolic compounds were detected showed higher antioxidant activity than all four samples from *V. jacquinii*. These results are consistent with many previous studies that have shown the antioxidant activity of phenolic compounds. Although phenolic compounds were not detected by HPLC in our hydrosol samples, these compounds have been reported in hydrosols of other plant species such as *Rosa damascena*, where Ulusoy et al. detected tocopherol and carotene [41]. Vlachou et al. studied several plant species to possibly use them as agricultural by-products, and they discovered some valuable compounds in hydrosol extracts of the barks of *Pinus* and *Eucalyptus* species, such as catechin, epicatechin, taxifolin and phenolic acids [42]. From these and many other studies, it can be concluded that hydrosols are valuable by-products of essential oil production and have many potentials in food preservation and industry in the future (e.g., prevention of biofilm formation on utensils and surfaces, natural antimicrobial agents in food industry) [17].

### 2.5. Antioxidant Activity

The antioxidant activity of the specialized metabolites has been only partially studied for this genus, but all previous studies showed good antioxidant properties, especially for iridoids and phenols. Harput et al. compared the bioactivity of chemical compounds in four species of the genus *Veronica*. Their study showed that a plant with higher phenolic content (*V. officinalis*) also had better antioxidant properties against DPPH [10] while in another study they found that *V. chamaedrys* had significant antioxidant activity against superoxide and *V. officinalis* against DPPH and nitric oxide [43]. In their another study DPPH activity was detected for water extracts of *V. cymbalaria*, *V. hederifolia*, *V. pectinata*, *V. persica* and *V. polita* species. The highest activity was detected for *V. polita* [44]. From Table 3, it can be seen that the results of antioxidant activity for EOs and hydrosols differ when comparing two methods (ORAC and DPPH). If we look at the results for ORAC method, highest activity showed St sample (6.6 ± 0.47 µmol/g of EO) which is not the case for results obtained with DPPH method. In this method Mr sample showed the highest activity (IC_50_ 246.55 ± 14.19 mg/mL). Results for hydrosols showed that GJ sample had the highest activity when using ORAC method (1.41 ± 0.149 µmol/g of hydrosol). The highest activity using the DPPH method showed Br sample of hydrosol (7.263 ± 0.593 mg/mL). When EOs and hydrosols are compared, it can be seen that EOs show higher activity in ORAC method while hydrosols show higher activity in DPPH method. Compared to our previous research on *V. saturejoides* ssp. *saturejoides* EOs of *V. jacquinii* have lower antioxidant activity in both methods [12]. Considering the study of Aazza et al. [45] who investigated the ORAC activity of hydrosols of several medicinal plants (*Lavandula officinalis*, *Origanum majorana*, *Rosmarinus officinalis*, *Salvia officinalis*, *Thymus vulgaris*, *Cinnamomum verum* and *Syzygium aromaticum*), the investigated *Veronica* hydrosols in the concentration of the volatiles of 10 mg/mL from all four locations have higher ORAC antioxidant activity than *S. officinalis*, *L. officinalis*, *R. officinalis* and *C. verum* hydrosol. A review of antioxidant research on other specialized metabolites from the genus *Veronica* (phenolic compounds and iridoid glycosides) shows that many of them have significant antioxidant activity [12].

In our previous research, we tested the antioxidant activity of the most abundant compound in the EOs, hexahydrofarnesyl acetone. This compound did not show antioxidant activity, so it was concluded that probably the antioxidant activity comes from another compound in EO or is the result of a synergistic effect between different compounds [12]. The results of the current research are consistent with these findings, as it can be seen that there are large differences in the activity of the EOs from the *V. austriaca* ssp. *jacquinii* and *V. saturejoides*, although the same major components are present in both oils. On the other hand, it can be inferred from this and previous research, that one compound which is more abundant in the hydrosols than in EOs may have an impact on the antioxidant activity of these extracts. The compound trans-p-mentha-1(7),8-dien-2-ol is present in all four samples of *V. jacquinii* hydrosols. This compound is not present in the EOs of *V. jacquinii* so this could be the reason for higher activity of the hydrosols in DPPH method. This compound is also present in the EO sample of *V. saturejoides*, which showed higher antioxidant activity than all other samples tested (from this study and from the other location of the same species from the previous study). Looking at the chemical structure of trans-p-mentha-1(7),8-dien-2-ol, it is an isomer of carveol which according to Kaur et. al. has high antioxidant activity potential [46]. They tested the antioxidant activity of carveol by four methods and concluded that the high antioxidant activity of carveol probably comes from an unsaturated hydroxyl group [46]. Looking again at the chemical structure of trans-p-mentha-1(7),8-dien-2-ol [47], we can see that this compound also has this hydroxyl group. Therefore, in future research, this compound should be tested for its antioxidant activity to see if this hypothesis is true. The other compound that could be the reason for the antioxidant activity of hydrosols is methyl eugenol which is major compound for all four samples of the tested hydrosols, especially in Br sample that showed the highest antioxidant activity in DPPH method and in GJ sample that shoed the highest activity in ORAC method.

The final activity is probably result of synergistic activity of the compounds present in EOs and hydrosols. Synergistic activity has been experimentally demonstrated in some studies. Amorati et al. found that the total oil tested on antioxidant activity had a higher activity than isolated single active compounds [48]. In their experiments, Huang et al. confirmed the synergistic effect of the minor components of cinnamon EO against *S. pullorum* [49]. In our previous studies on *V. spicata*, it was shown that there can also be a negative correlation between the amount of phenolic compounds and antioxidant activity [14]. This finding favors the possibility that other compounds (terpenoids and proteins) have an impact on the antioxidant activity. The results also show the importance of using different methods when evaluating antioxidant activity of plant extracts as different methods showed the different extract with the highest activity. This is expected as different compounds have different reactions with the compounds and radicals used in antioxidant methods [45].

### 2.6. Antiproliferative Activity of Essential Oils and Hydrosols

**Figure 5:**
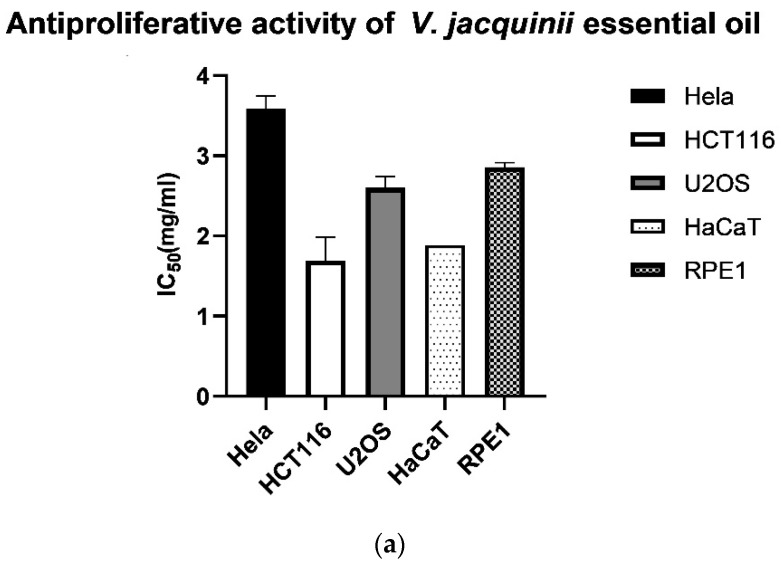

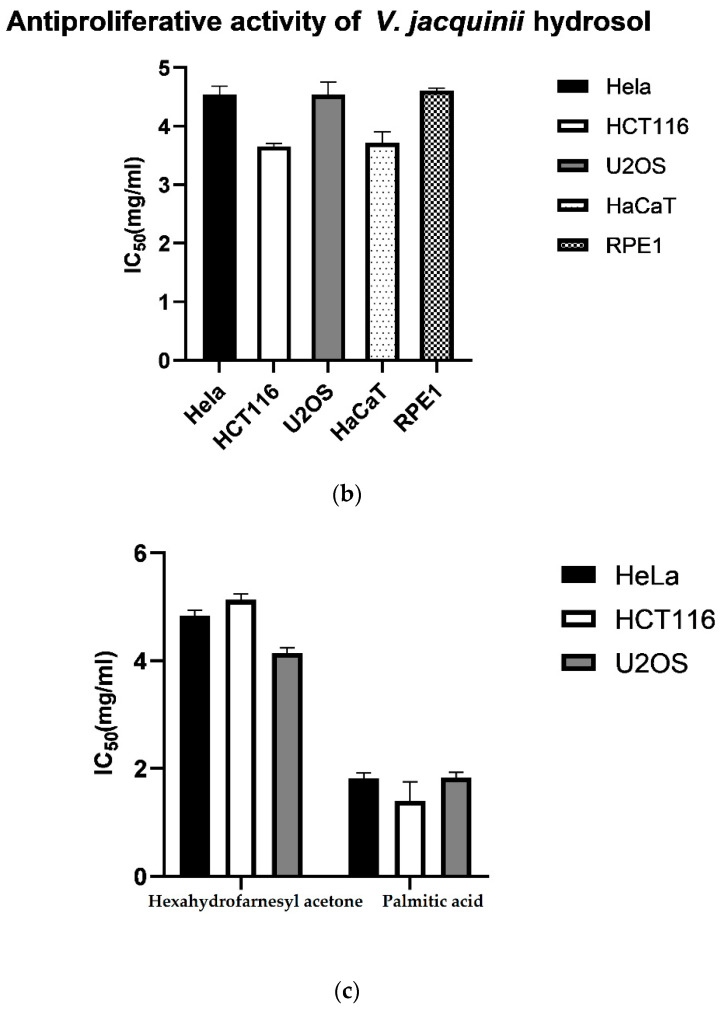
Antiproliferative activity of: (**a**) *V. austriaca* ssp. *jacquinii* EO; (**b**) *V. austriaca* ssp. *jacquinii* hydrosol; (**c**) Standards of the most abundant compounds.

Cytotoxic effect of the essential oil and hydrosol of *V. austriaca* ssp. *jacquinii* was tested on three cancer cell lines (HeLa, U2OS and HCT116) and two healthy cell lines (HaCaT and RPE1). The results showed that neither the EO nor the hydrosol exhibited toxicity to both cancer cells and healthy human cell lines (Figure 5a,b). The IC_50_ values were extremely high, over 1.5 mg/mL for the oil and over 3 mg/mL for the hydrosol. HeLa cells exhibited the highest resistance, requiring 3.47 mg/mL of the EO and 4.55 mg/mL of the hydrosol to inhibit cell growth by 50%. The colon (HCT116) and osteosarcoma (U2OS) cells showed slightly lower but still significant resistance to the effects of EO and hydrosol, with IC_50_ values of 1.9 mg/mL and 2.7 mg/mL for the EO and 3.65 and 4.54 mg/mL for the hydrosol, respectively. The EO and hydrosol also did not prevent the division of the healthy cells. The RPE1 cells, representing the epithelial cell model, were not sensitive to the effect of the oil and hydrosol with IC_50_ values of 2.82 and 4.61 mg/mL, respectively. In addition, the oil and hydrosol showed no toxicity to human keratinocytes (HaCaT) cells (IC_50_ values were 1.93 mg/mL for the EO and 3.72 mg/mL for the hydrosol). Two major compounds of the EO, hexahydrofarnesyl acetone and palmitic acid, were also tested on the antiproliferative activity. As expected, given the results on the cytotoxic activity of the EO, none of the compounds tested showed cytotoxic activity against cancer and healthy cell lines. The IC_50_ values for hexahydrofarnesyl acetone were above 4 mg/mL and for palmitic acid much lower, but still high enough not to inhibit proliferation of both cancer and healthy cells (IC_50_ > 1 mg/mL) (Figure 5c).

Various extracts from the aerial parts of *Veronica* species are used in folk medicine worldwide to treat many types of cancer [4]. Nevertheless, very few *Veronica* species have been studied for their cytotoxic activity in vitro and in vivo. Mainly methanolic and aqueous extracts of different *Veronica* species have been tested on cancer cell lines. The methanol extract of *V. cymbalaria*, *V. hederifolia*, *V. pectinata* var. *glandulosa*, *V. persica* and *V. polita* showed significant cytotoxic activity against KB epidermal carcinoma and melanoma cells. The isolated chloroform fraction showed dose dependent cytotoxicity [44]. The methanolic extract of the edible species *V. americana* showed cytotoxic activity against HF-6 (colon) and PC-3 (prostate) cancer cell lines [50]. The aqueous extract of *V. cuneifolia* subsp. *cuneifolia* and *V. cymbalaria* inhibited the division of Hep-2RD and L-20B cancer cells more than the healthy cell line, Vero cell line [51]. Flavonoids (Vtfs) isolated from *V. sibirica* stopped the division of MCF-7 (breast) cancer cells, the IC_50_ value was 42 ug/mL. The mechanism of inhibition of cancer cell division involved apoptosis, which was concentration dependent [52].

Considering the results obtained in the present study, the essential oil and hydrosol of *V. austriaca* ssp. *jacquinii* can be considered as a safe natural product.

### 2.7. Polyphenol Analysis in Dry Plant Material

Polyphenols are natural compounds that help the plant to protect itself from the ultraviolet radiation or aggression by pathogens [53]. Due to their good antioxidant, anti-neurodegenerative and anticancer properties, they are commonly consumed in the diet [54]. Table 4 shows the results for the polyphenol analyses *V. austriaca* ssp. *jacquinii* from four locations in Croatia. The highest content was found for total phenolic acids (TPA) in *V. austriaca* ssp. *jacquinii* from the Location Br while the yield of total flavonoids (TF) was found to be very low and similar for all investigated specimens of the studied species. Harput et al. conducted a study on a few *Veronica* species and found that total phenolic content (TP) was 200.20 mg/g in *V. officinalis* L., 139.92 mg/g in *V. peduncularis* M. Bieb., 127.64 mg/g in *V. orientalis* Mill., and 83.15 mg/g in *V. baranetzkii* Bordz. [10]. In the previous research of our team, species *V. spicata* had higher quantities of TP from methanol extracts than *V. austriaca* ssp. *jaquinii* (129.43 mg/g from dry weight of flowers and 141.28 mg/g from dry weight of leaves) [14]. In another study on the species *V. saturejoides* ssp. *saturejoides*, the TP content from methanol extracts was slightly higher in the Kamešnica sample (86.9 mg/g from dry weight of plant material) and similar in the Prenj sample (70.9 mg/g dry weight of plant material) compared to *V. austriaca* ssp. *jacquinii* (Table 4) [12].

## 3. Materials and Methods

### 3.1. Plant Material

**Figure 6:**
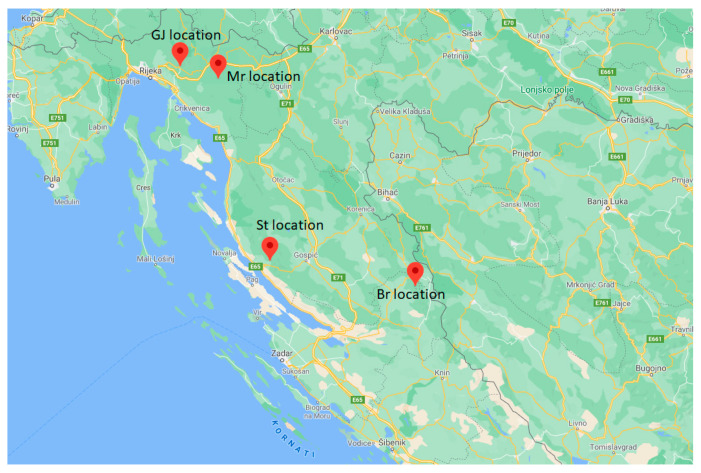
Map with the places of collection of the plant material.

Plant material for *V. austriaca* ssp. *jacquinii* was collected from four locations in Croatia (Table 5, Figure 6). Voucher specimens were deposited in the “Fran Kušan” hebarium (HFK-HR), Faculty of Pharmacy and Biochemistry, University of Zagreb, Croatia. For GC-MS analyses samples were air dried in a single layer and protected from direct sunlight for three weeks. Dried plant material was placed in double paper bags labeled with the sample number and stored in a dry place protected from light until analysis.

For micromorphological investigations of trichomes samples of seven plants were fixed in FAA (formalin/96% ethanol/acetic acid/water—5/70/5/20). After three days, samples were transferred to 70% ethanol.

### 3.2. Extraction of Volatiles from Hydrosols

After the extraction of EOs and hydrosols with Clevenger type apparatus, in order to determine antioxidant activity of hydrosols and compare it with EO antioxidant activity, volatiles from the hydrosols have been extracted with pentane/diethyl ether mixture. 5 mL of each hydrosol was extracted in a separation funnel with the mixture of 3 mL of pentane and 3 mL of diethyl ether. The organic layer was separated and dried over the anhydrous sodium sulphate. To calculate the exact concentration of hydrosol the organic solvent was evaporated. The volatile compounds that remained were weighed and the concentration for each hydrosol sample was calculated and expressed in mg/mL of hydrosol for the antioxidant activity. In the Table 6 masses of dry plant material and calculated yields of EOs and hydrosols are presented.

### 3.3. GC and GC-MS Analyses

Dried above ground parts (25—50 g) for each sample were hydrodistilled for 3 h in Clevenger type apparatus. For each sample (three separate extractions for each) volatile compounds and water residues (hydrosols) were collected. Both phases were analyzed with gas chromatography and mass spectrometry (GC-MS) according to a method described in our recent research [12]. GC was performed by gas chromatograph (model 3900, Varian Inc., Lake Forest, CA, USA) that is supplied with a flame ionization detector (FID), mass spectrometer (model 2100T; Varian Inc.), non-polar capillary column VF-5ms (30 m × 0.25 mm inside diameter, coating thickness 0.25 µm, Palo Alto, CA, USA) and polar capillary column CP-Wax 52 CB (30 m × 0.25 mm i.d., coating thickness 0.25 µm, Palo Alto, CA, USA). The chromatographic conditions for the analysis of lipophilic fraction (essential oils) were FID detector temperature 300 °C, injector temperature 250 °C. The gas carrier was helium at 1 mL min^−1^. The conditions for the VF-5ms column were temperature 60 °C isothermal for 3 min, and then increased to 246 °C at a rate of 3 °C min^−1^, and held isothermal for 25 min. Conditions for the CP Wax 52 column were: temperature 70 °C isothermal for 5 min, and then increased to 240 °C at a rate of 3 °C min^−1^ and held isothermal for 25 min. The injected volume was 2 µL and the split ratio was 1:20. The MS conditions were: ion source temperature 200 °C, ionization voltage 70 eV, mass scan range 40–350 mass units [14]. The individual peaks for all samples were identified by comparison of their retention indices of *n*-alkanes to those of authentic samples and literature [24,55], comparing it to our libraries from previous work [12,14] and to other previously published material for *Veronica* species [9,21,22,23]. The results are expressed as the mean value of three analyses with standard deviation.

### 3.4. Micromorphological Traits

For SEM-investigation, stem, leaf and calyx samples were transferred from 70% ethanol to 70% acetone, then further dehydrated (70%, 90% and 100% acetone) and subjected to critical point drying using CO_2_ as the drying medium (CPD030; Baltec). After that, samples were sputter coated with gold (Sputter Coater, AGAR) and examined under the scanning electron microscope XL30 ESEM (FEI) with 20 kV acceleration voltages in high vacuum mode [56]. Common terminology [30] was used in the description of trichomes.

### 3.5. Phenolic Compounds in Hydrosols

The phenolic compounds of the hydrosols were separated by high-performance liquid chromatography (HPLC) on a Ultra Aqueous C18 column (250 × 4.6 mm, 5 mm; Restek; Bellefonte, PA, USA) according to a method described in Nazlić et al. [12]. Gradient chromatography conditions were set according to the methodology by Jukić Špika et al. [57]. Solvents used for mobile phase were 0.2% phosphoric acid (A), methanol (B) and acetonitrile (C). At 0 min, the gradient was 96% A, 2% B, and 2% C. During the first 40 min, ratios were changed from the initial value to 50% A, 25% B, and 25% C and were subsequently changed to 40% A, 30% B, and 30% C from 40 to 45 min. The gradient was changed to 50% B and 50% C from 45 to 60 min, and kept at these values until 70 min. The obtained solvent ratio was retained for the last 10 min to achieve the stability of the column to the initial conditions.

The standard and solvents were of analytical grade and were purchased from Sigma-Aldrich (Sigma-Aldrich, St. Louis, MO, USA). In the preparation of all solvents deionized water (Milli-Q) was used.

### 3.6. Polyphenol Analysis

#### 3.6.1. Apparatus and Chemicals

##### Apparatus

Water-bath, reflux condenser, UV/Vis spectrophotometer Agilent 8453 (Agilent, Karlsruhe, Germany) with PC-HP 845x UV-Visible System (Agilent, Karlsruhe, Germany) and 1 cm quartz cells were used for all absorbance measurements. Filtration of prepared sample solutions was performed by using 0.20 µm Minisart-plus membrane filter (Sartorius AG, Goettingen, Germany).

##### Reagents and solutions (TP/T)

Pro analysi chemicals, as well as double distilled water were used throughout the work. The solution of 30% methanol (Kemika, Zagreb, Croatia) was used for plant material extraction. The solution of 33% sodium carbonate decahydrate (Kemika, Zagreb, Croatia) was used for sample preparation.

Except for the Folin–Ciocalteu phenol reagent (FCR), casein (Merck, Darmstadt, Germany) and quercetin (Roth, Karlsruhe, Germany), all chemicals and reagents for polyphenol analysis were of analytical quality grade and were supplied by Kemika (Zagreb, Croatia).

#### 3.6.2. Total Polyphenol and Tannin Analysis (Folin–Ciocalteu Phenol Reagent (FCR) Procedure)

Total polyphenols (TP) and tannins (T) in aboveground parts of *V. austriaca* ssp. *jacquinii* were determined by using the prevalidated *FCR procedure* for polyphenols analysis according to Jurišić Grubešić et al. [58].

The finely ground above-ground plant portions (0.25 g) were extracted with 80 mL of 30% methanol in a reflux flask, heating on a boiling water bath for about 15 min. After cooling, the extract was filtered into a 100.0 mL volumetric flask and made up to the mark with 30% (*v/v*) methanol. 2.0 mL of the filtrate was mixed with 8.0 mL of distilled water and 10.0 mL of sodium acetate solution (1.92 g of sodium acetate trihydrate and 0.34 mL of acetic acid were mixed and made up to 100.0 mL with distilled water). The buffer solution maintains a constant pH value of the medium (pH = 5), which is optimal for the precipitation of tannins. The solution obtained as described was labeled as solution 1 (S1). 10.0 mL of S1 was shaken with 50 mg of casein on a shaker for 45 min. The solution was filtered, and the resulting filtrate was solution 2 (S2).

1.0 mL of S1 and S2 were mixed separately in 10.0 mL volumetric flasks with 0.5 mL of Folin-Ciocalteu’s phenolic reagent and made up to the mark with 33% (*m/v*) sodium carbonate decahydrate solution. The absorbances of the obtained blue solutions were measured at 720 nm, with distilled water as a blank. The value given by S1 corresponds to the content of TP, while the difference between the values obtained for S1 and S2 represents the content of T bound to casein.

A calibration curve was developed using tannin as a standard substance. For this purpose, 10 mg of tannin was dried at 80 °C and dissolved in 100.0 mL of distilled water (standard stock solution). The working standard was prepared by mixing 5.0 mL of standard stock solution and 5.0 mL of buffer solution. The concentration range, obtained by diluting the volume from 0.2 to 1.2 mL of working standard to 10.0 mL with buffer solution (corresponding to a tanning concentration of 0.001 to 0.006 mg/mL), gives a linear increase in absorbance.

For the measured absorbance values of S1 and S2, the corresponding concentrations from the calibration diagram are obtained. The difference between the TP content (obtained by measuring S1), and the content determined for S2, represents the content of tannins precipitated with casein [59].

The contents of TP and T in *V. austriaca* ssp. *jacquinii* extracts were evaluated in three independent analyses and were expressed as mg/g of dry weight of herbal material according to following equation:

TP (mg/g) = A_S1_/0.0025

T (mg/g) = (A_S1_/0.0025) − (A_S2_/0.0025)

A_S1_, A_S2_ = measured absorbance of S1 and S2, respectively.

#### 3.6.3. Total Flavonoid (TF) Analysis—TF Procedure

TF Procedure was performed as follows: 0.200 g of powdered herbal drug is extracted individually for 30 min with 20 mL of acetone, 2 mL of 25% hydrochloric acid and 1 mL of 0.5% hexamethylenetetramine solution, heating to reflux in a water bath. The hydrolysate was passed through a cotton ball, and the drug residue on the cotton wool was re-extracted with 20 mL of acetone, heating to boiling for 10 min. This solution was also passed through a cotton ball, and the acetone extraction described above was repeated a total of 3 times. The combined filtrates were diluted with acetone to 100.0 mL.

20.0 mL of the hydrolysate was mixed with 20 mL of water and then extracted first with 15 mL and three times with 10 mL each of ethyl acetate. The combined ethyl acetate phases were washed twice with 40 mL each of water, passed through cotton wool and diluted with ethyl acetate to 50.0 mL. Two portion of 10.0 mL of this solution were transferred to two 25.0 mL volumetric flasks. To each flask was added 0.5 mL of 0.5% aqueous sodium citrate solution. An additional 2 mL of aluminum chloride solution was added to one flask (2 g of aluminum chloride hexahydrate was dissolved in 100.0 mL of 5% methanolic acetic acid solution). Both flasks were then made up to 25.0 mL with 5% methanolic acetic acid solution. After 45 min, the absorbances of the solutions with aluminum chloride were measured at 425 nm, in a 1 cm thick layer. The compensating solution was a previously prepared solution without aluminum chloride.

The mass concentration of flavonoids is calculated as quercetin, according to the expression:

TF (%) = A × 0.772/b (A = absorbance; b = drug weight expressed in g).

#### 3.6.4. Determination of Total Phenolic Acids (TPA) (TPA Procedure)

TPA Procedure in selected plant samples was performed by spectrophotometric method prescribed by the European Pharmacopoeia [60].

In 0.200 g of powdered herbal drug was added 80 mL of 50% ethanol and heated in a reflux flask on a boiling water bath for 30 min. After cooling and filtration, the filtrate was made up to 100.0 mL with 50% ethanol in a volumetric flask (stock solution). The test solution was prepared by mixing 1.0 mL of stock solution with 2.0 mL of 0.5 M hydrochloric acid, 2.0 mL of solution obtained by dissolving 10 g of sodium nitrite and 10 g of sodium molybdate in 100.0 mL of water and 2.0 mL 8.5% sodium hydroxide solution. The flask was made up to 10.0 mL with distilled water. The compensating solution was obtained by diluting 1.0 mL of stock solution with water to 10.0 mL. The absorbance of the resulting solutions was immediately measured at 505 nm (expressed as rosmarinic acid) and 525 nm (expressed as chlorogenic acid).

The mass fraction of phenolic acids calculated and expressed as rosmarinic acid (TPA1):

TPA1 (%) = A × 2.5/m

where A is the measured absorbance at 505 nm, taking the specific absorbance of rosmarinic acid at 505 nm to be 400; m—mass of the drug expressed in grams.

The mass fraction of phenolic acids calculated and expressed as chlorogenic acid (TPA2):

TPA2 (%) = A × 5.3/m

where A is the measured absorbance at 525 nm, taking the specific absorbance of chlorogenic acid at 525 nm to be 188; m—mass of the drug expressed in grams.

### 3.7. Antioxidant Activity of Essential Oils and Hydrosols

#### 3.7.1. ORAC

The assay was performed in a Perkin–Elmer LS55 spectrofluorimeter, using 96-well white polystyrene microtiter plates (Porvair Sciences, Leatherhead, UK) according to a method described by Fredotović et al. [61], with some adjustments due to different extracts described in our previous research [12]. All measurements were performed in triplicate by a method described in Nazlić et. al. [12].

#### 3.7.2. DPPH

The antioxidant capacity of the extracts was assessed by the DPPH method previously described by Mensor et al. and Payet et al. [62,63] and adjusted to our plant extracts as described in the method from our previous research [12]. Calculation and expressing the results were carried out according to the method described in our previous research in Nazlić et al. [12].

### 3.8. Cell Culture

We received three cancer cell lines: Cervical Cancer Cell Line (HeLa), Human Colon Cancer Cell Line (HCT116) and Human Osteosarcoma Cell Line (U2OS), and two healthy cell lines: Human Epidermal Keratinocyte Line (HaCaT) and Retinal Pigmented Epithelial Cells (RPE1) as a gift from Professor Janoš Terzić from the School of Medicine, University of Split. Cells were grown in an incubator under humidified conditions with 5% CO_2_ and 37 °C, in a Dulbecco’s modified Eagle’s medium (DMEM Euroclone, Milano, Italy) containing 4.5 g/L glucose, 10% fetal bovine serum (FBS), and 1% antibiotics (penicillin and streptomycin, EuroClone).

### 3.9. Cell Proliferation Assay

The antiproliferative capacity of the EO of *V. austriaca* ssp. *jacquinii* was determined on HeLa, HCT116 and U2OS cancer cells and in healthy cells HaCaT and RPE1 using the MTS-based CellTiter 96^®^ Aqueous Assay (Promega, Madison, WI, USA). Cells were grown in an incubator at 37 °C and 5% CO_2_ until they reached 80% confluence. They were counted using a handheld automated cell counter (Scepter, Merck), and 5000 cells/well were seeded in 96-well plates with a serial dilution of the EOs and hydrosols. The cells were then cultured for an additional 48 h. Thereafter, 20 μL MTS tetrazolium reagent (Promega, Madison, WI, USA) was added to each well and left in the incubator for a further 3 h. Then, absorbance was measured at 490 nm using a microplate reader (Bio-Tek, EL808). IC_50_ values were calculated from three independent experiments using GraFit 6 data analysis software (Erithacus, East Grinstead, UK).

### 3.10. Statistical Analyses

Statistical analysis was performed in GraphPad Prism Version 9 (GraphPad Software, San Diego, CA, USA). All data are expressed as mean ± SD (*n* ≥ 3). Data included in the PCA analyses was data obtained from the GC-MS analyses. The statistical significance for free volatile compounds, total phenolic compounds and antioxidant activity was assessed by 2way ANOVA followed by Šídák’s multiple comparisons test and Tukey’s multiple comparison test, *p* < 0.05. Statistical tests were performed separately for lipophilic (essential oils) and hydrophilic fractions (hydrosols).

## 4. Conclusions

In this study, the free volatile compounds of the species *V. austriaca* ssp. *jacquinii* and their biological activity were reported. The volatile compounds extracted in essential oils and hydrosols from four locations were compared. Hexahydrofarnesyl acetone and hexadecanoic acid were the major components of the essential oils from all four locations. The major components of the hydrosols were methyl eugenol and thymol. Our research has shown that hydrosols can exhibit stronger antioxidant activity than essential oils, even though they contain fewer dissolved molecules in them. As they showed no toxicity towards healthy cells they could be used as a safe product in cosmetics or for food preservation. Essential oils and hydrosols from the genus *Veronica* are just beginning to be studied in more detail as other compounds were more investigated for this genus, such as iridoids and phenolic compounds. We believe that the free volatile compounds from this genus are also valuable resources and should be further investigated in the future.

## Figures and Tables

**Table 1:** Chemical composition of the essential oil from four locations from aerial parts of *Veronica austriaca* ssp. *jacquinii*.

			Mr	St	Br	GJ
Component	RI^1^	RI^2^	EO ± SD (%)	EO ± SD (%)	EO ± SD (%)	EO ± SD (%)
Sesquiterpene hydrocarbons			1.46	7.68	2.78	2.57
*E*-Caryophyllene *	1424	1585	0.31 ± 0.01 ^d^	2.35 ± 0.01 ^a^	1.52 ± 0.01 ^c^	2.15 ± 0.01 ^b^
*δ*-Cadinene	1517	1745	1.15 ± 0.01 ^b^	1.84 ± 0.01 ^a^	0.99 ± 0.02 ^c^	0.42 ± 0.01 ^d^
*allo*-Aromadendrene	1465	1662	-	0.88 ± 0.01	-	-
*β*-Chamigrene	1476	1724	-	-	0.27 ± 0.02	-
Germacrene D	1482	1692	-	2.61 ± 0.05	-	-
Oxygenated sesquiterpenes			53.01	30.93	29.26	23.88
Spathulenol	1577	2101	-	1.84 ± 0.01 ^a^	0.45 ± 0.01 ^c^	0.54 ± 0.01 ^b^
*β*-Caryophyllene oxide *	1581	1955	0.45 ± 0.01 ^b^	0.62 ± 0.01 ^a^	0.48 ± 0.01 ^b^	-
*γ*-Eudesmol	1632	2175	-	0.25 ± 0.03	-	-
α-Bisabolol oxide	1748	2511	-	0.37 ± 0.01	-	-
Hexahydrofarnesyl acetone	1839	2113	52.56 ± 0.01 ^a^	27.85 ± 0.01 ^c^	28.33 ± 0.01 ^b^	23.34 ± 0.01 ^d^
Phenolic compounds			0.63	2.68	0.84	0.81
Methyl eugenol	1403	2005	-	1.26 ± 0.01	-	-
*(Z)*-Methyl isoeugenol	1451	2070	0.63 ± 0.03 ^c^	1.42 ± 0.01 ^a^	0.84 ± 0.01 ^b^	0.81 ± 0.01 ^b^
Acids, alcohols and esters			35.5	47.69	57.74	62.52
1-Hexadecanol	1874	2371	-	0.57 ± 0.03	-	-
Hexadecanoic acid	1959	2912	26.71 ± 0.02 ^d^	47.12 ± 0.01 ^c^	54.53 ± 0.05 ^b^	58.91 ± 0.03 ^a^
Oleic acid	2133	2998	2.35 ± 0.01 ^a^	-	0.51 ± 0.03 ^b^	-
Octadecanol acetate	2209	2211	6.24 ± 0.01 ^a^	-	2.26 ± 0.01 ^c^	3.61 ± 0.01 ^b^
1-Heptatriacotanol	2309	2309	-	-	0.44 ± 0.01	-
Hydrocarbons			1.63	2.03	2.51	2.05
Eicosane *	2000	2000	-	1.16 ± 0.01 ^b^	0.45 ± 0.01 ^c^	1.24 ±0.04 ^a^
Heneicosane *	2100	2100	0.53 ± 0.02 ^a^	0.35 ± 0.01 ^b^	-	0.53 ± 0.01 ^a^
Docosane *	2200	2200	0.38 ± 0.01 ^c^	0.29 ± 0.01 ^c^	0.81 ± 0.01 ^a^	-
Tricosane *	2300	2300	-	-	0.63 ± 0.01	-
Tetracosane *	2400	2400	-	0.23 ± 0.01 ^c^	0.62 ± 0.01 ^a^	0.28 ± 0.01 ^b^
Pentacosane *	2500	2500	0.72 ± 0.04	-	-	-
Total identification (%)			92.03	91.01	93.13	91.83

Retention indices (RI) were determined relative to a series of *n*-alkanes (C8–C40) on capillary columns VF5-ms (RI^1^) [24] and CPWax 52 (RI^2^) [25]; Identification method: RI, comparison of RIs with those listed in a homemade library, reported in the literature [24], and/or authentic samples; comparison of mass spectra with those in mass spectral libraries NIST02 and Wiley 9; * co-injection with reference compounds; %—relative peak area; SD, standard deviation. Significant differences for every volatile compound present in more than one location were determined 2way ANOVA followed by Šídák’s multiple comparisons test. ^a,b,c,d^—Mean values in the same row with different superscript letters indicate a statistically significant difference between data from four locations (*p* < 0.05).

**Table 2:** Chemical composition of the hydrosols from four locations from aerial parts of *Veronica austriaca* ssp. *jacquinii*.

			Mr	St	Br	GJ
Component	RI^1^	RI^2^	Hy ± SD (%)	Hy ± SD (%)	Hy ± SD (%)	Hy ± SD (%)
Monoterpene hydrocarbons			1.56	-	-	-
α-Thujene	924	1032	0.68 ± 0.01	-	-	-
*β*-Phellandrene	1002	1194	0.88 ± 0.03	-	-	-
Oxygenated monoterpenes			21.23	16.87	8.02	15.01
*trans*-Linalool oxide *	1088	1434	0.36 ± 0.04	-	-	-
*n*-Nonanal	1100	1389	4.35 ± 0.01 ^a^	2.82 ± 0.01 ^b^	-	-
Borneol	1176	1719	1.56 ± 0.01 ^a^	0.51 ± 0.01 ^b^	-	-
Camphor	1151	1499	2.18 ± 0.01 ^b^	0.92 ± 0.01 ^c^	-	3.53 ± 0.01 ^a^
Pinocarvone	1160	1565	2.00 ± 0.01	-	-	-
*trans*-*p*-Mentha-1(7),8-dien-2-ol	1187	1803	7.69 ± 0.01 ^a^	5.24 ± 0.01 ^d^	7.44 ± 0.01 ^b^	6.37 ± 0.02 ^c^
Hexyl 2-methyl butanoate	1233	1425	1.26 ± 0.01 ^c^	3.12 ± 0.03 ^b^	-	4.36 ± 0.01 ^a^
Menthyl acetate	1294	1550	1.83 ± 0.03 ^b^	4.26 ± 0.01 ^a^	0.58 ± 0.01 ^d^	0.75 ± 0.06 ^c^
Sesquiterpene hydrocarbons			6.87	3.69	1.59	6.62
*E*-Caryophyllene *	1424	1585	2.65 ± 0.01 ^a^	1.33 ± 0.01 ^b^	0.66 ± 0.02 ^d^	0.73 ± 0.01 ^c^
δ-Cadinene	1517	1745	2.36 ± 0.01 ^a^	-	0.93 ± 0.06 ^b^	2.38 ± 0.08 ^a^
*allo*-Aromadendrene	1465	1662	1.52 ± 0.01 ^a^	-	-	1.24 ± 0.01 ^b^
*β*-Chamigrene	1478	1724	0.34 ± 0.01	-	-	-
Germacrene D	1482	1692	-	2.36 ± 0.01 ^a^	-	2.27 ± 0.01 ^b^
Oxygenated sesquiterpenes			3.20	9.49	9.60	4.26
Spathulenol	1577	2101	-	-	-	1.23 ± 0.01
*β*-Caryophyllene oxide *	1581	1955	2.18 ± 0.01 ^a^	1.27 ± 0.01 ^b^	1.10 ± 0.01 ^c^	0.50 ± 0.01 ^d^
γ-Eudesmol	1632	2175	-	-	-	-
α-Muurolol	1645	2181	-	1.23 ± 0.01	-	-
α-Cadinol	1655	2208	-	2.45 ± 0.01	-	-
α-Bisabolol	1685	2210	0.54 ± 0.03 ^b^	-	0.50 ± 0.01 ^b^	1.32 ± 0.01 ^a^
α-Bisabolol oxide	1748	2511	-	-	0.30 ± 0.01 ^b^	0.51 ± 0.01 ^a^
Hexahydrofarnesyl acetone	1839	2113	0.48 ± 0.01 ^d^	4.54 ± 0.04 ^b^	7.70 ± 0.02 ^a^	0.70 ± 0.01 ^c^
Phenolic compounds			43.41	37.69	62.68	49.64
Thymol *	1289	2154	8.35 ± 0.05 ^b^	9.45 ± 0.02 ^a^	3.48 ± 0.01 ^d^	4.18 ± 0.01 ^c^
Thymol acetate	1349	-	3.66 ± 0.01 ^a^	2.27 ± 0.01 ^c^	-	2.43 ± 0.03 ^b^
Methyl eugenol	1403	2005	30.23 ± 0.02 ^c^	23.35 ± 0.01 ^d^	57.93 ± 0.01 ^a^	41.85 ± 0.01 ^b^
*(Z)*-Methyl isoeugenol	1451	2070	1.17 ± 0.01 ^c^	2.62 ± 0.06 ^a^	1.27 ± 0.01 ^b^	1.18 ± 0.01 ^c^
Acids, alcohols and esters			6.93	13.23	6.94	6.24
1-Hexadecanol	1874	2371	-	-	2.44 ± 0.01	-
Hexadecanoic acid	1959	2912	4.57 ± 0.01 ^b^	6.28 ± 0.02 ^a^	2.25 ± 0.01 ^c^	1.89 ± 0.01 ^d^
Oleic acid	2133	2998	0.28 ± 0.01 ^d^	4.85 ± 0.01 ^a^	0.46 ± 0.01 ^c^	3.79 ± 0.01 ^b^
Octadecanol acetate	2209	-	1.54 ± 0.01 ^a^	1.18 ± 0.01 ^b^	0.57 ± 0.02 ^c^	0.56 ± 0.01 ^c^
1-Heptatriacotanol	2309	2309	0.54 ± 0.01 ^c^	0.92 ± 0.01 ^b^	1.22 ± 0.01 ^a^	-
Hydrocarbons			11.25	11.96	3.77	9.62
Eicosane *	2000	2000	1.52 ± 0.04 ^a^	-	0.43 ± 0.01 ^c^	1.37 ± 0.01 ^b^
Heneicosane *	2100	2100	0.71 ± 0.01 ^a^	-	0.29 ± 0.01 ^c^	0.56 ± 0.06 ^b^
Docosane *	2200	2200	1.15 ± 0.01 ^a^	-	0.36 ± 0.01 ^b^	1.19 ± 0.01 ^a^
Tricosane *	2300	2300	0.63 ± 0.01 ^b^	0.85 ± 0.02 ^a^	-	-
Tetracosane *	2400	2400	-	0.48 ± 0.01 ^c^	0.87 ± 0.01 ^a^	0.68 ± 0.01 ^b^
Pentacosane *	2500	2500	0.67 ± 0.01 ^a^	0.25 ± 0.01 ^b^	-	-
Hexacosane *	2600	2600	2.54 ± 0.01 ^b^	3.08 ± 0.01 ^a^	0.97 ± 0.02 ^c^	0.83 ± 0.03 ^d^
Heptacosane *	2700	2700	3.14 ± 0.01 ^b^	3.22 ± 0.01 ^a^	0.29 ± 0.01 ^d^	1.03 ± 0.01 ^c^
Octacosane *	2800	2800	0.89 ± 0.01 ^c^	4.08 ± 0.01 ^a^	0.56 ± 0.02 ^d^	3.96 ± 0.01 ^b^
Total identification (%)			94.45	92.93	92.6	91.39

Retention indices (RI) were determined relative to a series of *n*-alkanes (C8–C40) on capillary columns VF5-ms (RI^1^) [24] and CPWax 52 (RI^2^) [25]; Identification method: RI, comparison of RIs with those listed in a homemade library, reported in the literature [24], and/or authentic samples; comparison of mass spectra with those in mass spectral libraries NIST02 and Wiley 9; * co-injection with reference compounds; %—relative peak area; SD, standard deviation. Significant differences for every volatile compound present in more than one location were determined using 2way ANOVA followed by Šídák’s multiple comparisons test. ^a,b,c,d^—Mean values in the same row with different superscript letters indicate a statistically significant difference between data from four locations (*p* < 0.05).

**Table 3:** Antioxidant potential of *V. austriaca* ssp. *jacquinii* of the essential oil and hydrosol determined by ORAC and DPPH method.

**Essential Oils**				
**Antioxidant Assay**	**1 (Mr)**	**2 (St)**	**3 (Br)**	**4 (GJ)**
ORAC (Trolox eq)	4.25 ± 0.42 ^a^	6.6 ± 0.47 ^a^	4.87 ± 0.49 ^a^	4.92 ± 0.38 ^a^
DPPH (Trolox eq)	0.135 ± 0.02 ^a^	0.06 ± 0.002 ^a^	0.09 ± 0.008 ^a^	0.02 ± 0.002 ^a^
DPPH (% inhibition)	2.22 ± 0.17 ^a^	1.16 ± 0.05 ^a^	1.51 ± 0.15 ^a^	0.48 ± 0.06 ^a^
DPPH (IC 50)	246.55 ± 14.19 ^a^	528.47 ± 17.57 ^c^	428.96 ± 21.88 ^b^	1138.4 ± 39.03 ^d^
**Hydrosols**				
**Antioxidant Assay**	**1 (Mr)**	**2 (St)**	**3 (Br)**	**4 (GJ)**
ORAC (Trolox eq)	1.24 ± 0.089 ^a^	0.884 ± 0.041 ^a^	1.33 ± 0.069 ^a^	1.41 ± 0.149 ^a^
DPPH (Trolox eq)	0.355 ± 0.019 ^a^	0.209 ± 0.017 ^a^	0.342 ± 0.026 ^a^	0.085 ± 0.008 ^a^
DPPH (% inhibition)	64.66 ± 3.61 ^b^	47.092 ± 3.67 ^a^	68.841 ± 5.623 ^b^	35.528 ± 3.532 ^c^
DPPH (IC 50)	7.73 ± 0.431 ^a^	10.617 ± 0.827 ^ab^	7.263 ± 0.593 ^a^	14.073 ± 1.4 ^b^

ORAC, oxygen radical absorbance capacity, results for EOs expressed as µmol of Trolox equivalents (TE) per g of EO (10 mg/mL) and for hydrosols as µmol of Trolox equivalents (TE) per g of the total (undiluted) tested hydrosol sample (10 mg volatiles/mL of hydrosol); DPPH, results for EOs expressed as µmol of Trolox per g of EO (10 mg/mL) and for hydrosols as µmol of Trolox per g of absolute hydrosol, IC50 expressed in mg/mL for EOs; SD = standard deviation of triplicate analysis; significant differences were determined using 2Way ANOVA followed by Tukey’s multiple comparison test. ^a,b,c,d^—Mean values in the same row with different superscript letters indicate a statistically significant difference between data from four locations (*p* < 0.05).

**Table 4:** Contents of total polyphenols (TP), total tannins (T), total flavonoids (TF), and total phenolic acids (TPA) in *V. austriaca* ssp. *jacquinii*.

Sample	TP (mg/g DW)	T (mg/g DW)	TF (mg/g DW)	TPA1 (505 nm) (mg/g DW)	TPA2 (525 nm) (mg/g DW)
1 (Mr)	55.98 ± 0.30 ^c^	8.29 ± 0.70 ^a^	1.29 ± 0.00 ^a^	15.68 ± 2.10 ^b^	19.91 ± 2.00 ^a^
2 (St)	70.60 ± 7.60 ^b^	9.06 ± 6.40 ^a^	2.10 ± 0.00 ^a^	20.07 ± 1.20 ^ab^	17.83 ± 1.20 ^a^
3 (Br)	78.79 ± 1.30 ^a^	8.98 ± 1.40 ^a^	2.05 ± 0.00 ^a^	26.58 ± 2.00 ^a^	24.16 ± 1.80 ^a^
4 (GJ)	66.55 ± 0.50 ^b^	8.58 ± 0.80 ^a^	1.47 ± 0.00 ^a^	19.47 ± 0.60 ^b^	17.38 ± 0.50 ^a^

Note: DW, dry weight; SD = standard deviation of triplicate analysis; significant differences were determined using Tukey’s multiple comparison test. ^a,b,c^—Mean values in each column with different superscript letters indicate a statistically significant difference between data from four locations (*p* < 0.05).

**Table 5:** Locations of the plant material collection (for volatile compounds extraction).

	Locality	Coordinates	Altitude a.s.l. (m)	Date of Collection	Abbrev.
1.	Mrkopalj	45°18′59″ N; 14°50′43″ E	820	June 2019	Mr
2.	Stupačinovo	44°32′21″ N; 15°09′51″ E	971	June 2019	St
3.	Lika, Brezovac	44 47′42″ N; 15°34′28″ E	798	June 2020	Br
4.	Gornje Jelenje	45°21′50″ N; 14°37′32″ E	880	May 2020	GJ

**Table 6:** Yield of obtained volatile compounds from EOs and hydrosols.

Location	Mass of Dry Plant Material Used for Isolations of Volatiles (g)	Mass of EO (mg)	Yield of EO (%)	Mass of Volatiles from Hy (mg)	Yield of Volatiles from Hy (%)
1 (Mr)	50	190	0.38	138	0.28
2 (St)	30	140	0.47	126	0.42
3 (Br)	35	180	0.51	104	0.30
4 (GJ)	25	160	0.64	113	0.45

## Supplementary Materials

The following are available online at https://www.mdpi.com/article/10.3390/plants10112529/s1. Table S1. Chemical composition of the essential oil from four locations from aerial parts of *Veronica austriaca* ssp. *jacquinii* calculated based on the masses of EOs extracted from dry plant material; Table S2. Chemical composition of the hydrosols from four locations from aerial parts of *Veronica austriaca* ssp. *jacquinii* calculated based on the masses of volatile compounds extracted from hydrosols from dry plant material; Figure S1. RIC chromatograms of the four samples of EOs of *V. jacquinii* with marked major compounds; Figure S2. RIC chromatograms of the four samples of hydrosols of *V. jacquinii* with marked major compounds.
