# Supplementary material for: Free Volatile Compounds of Veronica austriaca ssp. jacquinii (Baumg.) Eb. Fisch. and Their Biological Activity
Source: Plants (Basel). 2021 Nov 20;10(11):2529. doi: 10.3390/plants10112529 (PMC8618523; doi:10.3390/plants10112529)
Supplement: Supplementary file 1 [file plants-10-02529-s001.zip › plants-1459605-supplementary.pdf]

Table S1. Chemical composition of the essential oil from four locations from aerial parts of *Veronica austriaca* ssp. *jacquinii* calculated based on the masses of EOs extracted from dry plant material.

Component	RI[1]	RI[2]	Mr	St	Br	GJ
			**EO ± SD (mg/g dry plant material)**	**EO ± SD (mg/g dry plant material)**	**EO ± SD (mg/g dry plant material)**	**EO ± SD (mg/g dry plant material)**
**Sesquiterpene hydrocarbons**			**0.056**	**0.358**	**0.143**	**0.165**
E-Caryophyllene*	1424	1585	0.012 ± 0.00038	0.109 ± 0.00047	0.078 ± 0.00051	0.138 ± 0.00064
δ-Cadinene	1517	1745	0.044 ± 0.00038	0.086 ± 0.00047	0.051 ± 0.00103	0.027 ± 0.00064
allo-Aromadendrene	1465	1662	-	0.041 ± 0.00047	-	-
β-Chamigrene	1476	1724	-	-	0.014 ± 0.00103	-
Germacrene D	1482	1692	-	0.122 ± 0.00233	-	-
**Oxygenated sesquiterpenes**			**2.014**	**1.443**	**1.505**	**1.529**
Spathulenol	1577	2101	-	0.086 ± 0.00047	0.023 ± 0.00051	0.035 ± 0.00064
β-Caryophyllene oxide*	1581	1955	0.017 ± 0.00038	0.029 ± 0.00047	0.025 ± 0.00051	-
γ-Eudesmol	1632	2175	-	0.012 ± 0.0014	-	-
α-Bisabolol oxide	1748	2511	-	0.017 ± 0.00047	-	-
Hexahydrofarnesyl acetone	1839	2113	1.997 ± 0.00038	1.299 ± 0.00047	1.457 ± 0.00051	1.494 ± 0.00064
**Phenolic compounds**			**0.024**	**0.125**	**0.043**	**0.052**
Methyl eugenol	1403	2005	-	0.0588 ± 0.00047	-	-
(Z)-Methyl isoeugenol	1451	2070	0.024 ± 0.00114	0.066 ± 0.00047	0.043 ± 0.00051	0.052 ± 0.00064
**Acids, alcohols and esters**			**1.341**	**2.226**	**2.969**	**4.001**
1-Hexadecanol	1874	2371	-	0.027 ± 0.0014	-	-
Hexadecanoic acid	1959	2912	1.015 ± 0.00076	2.199 ± 0.000467	2.804 ± 0.00257	3.770 ± 0.00192
Oleic acid	2133	2998	0.089 ± 0.00038	-	0.026 ± 0.00154	-
Octadecanol acetate	2209	2211	0.237 ± 0.00038	-	0.116 ± 0.00051	0.231 ± 0.00064
1-Heptatriacotanol	2309	2309	-	-	0.023 ± 0.00051	-
**Hydrocarbons**			**0.06114**	**0.095**	**0.129**	**0.131**
Eicosane*	2000	2000	-	0.054 ± 0.00047	0.023 ± 0.00051	0.079 ± 0.00256
Heneicosane*	2100	2100	0.02014 ± 0.00076	0.016 ± 0.00047	-	0.034 ± 0.00064
Docosane*	2200	2200	0.014 ± 0.00038	0.014 ± 0.00047	0.042 ± 0.00051	-
Tricosane*	2300	2300	-	-	0.032 ± 0.00051	-
Tetracosane*	2400	2400	-	0.011 ± 0.00047	0.032 ± 0.00051	0.018 ± 0.00064
Pentacosane*	2500	2500	0.027 ± 0.00152	-	-	-
**Total identification (mg/g)**			**3.496**	**4.247**	**4.789**	**5.878**

Retention indices (RI) were determined relative to a series of *n*-alkanes (C8–C40) on capillary columns VF5-ms (RI[1]) [24] and CPWax 52 (RI[2]) [25]; Identification method: RI, comparison of RIs with those listed in a homemade library, reported in the literature [24], and/or authentic samples; comparison of mass spectra with those in mass spectral libraries NIST02 and Wiley 9; * co-injection with reference compounds; SD, standard deviation.

Table S2. Chemical composition of the hydrosols from four locations from aerial parts of *Veronica austriaca* ssp. *jacquinii* calculated based on the masses of volatile compounds extracted from hydrosols from dry plant material

	Mr	St	Br	GJ

Component	RI[1]	RI[2]	Hy ± SD (mg/g dry plant material)	Hy ± SD (mg/g dry plant material)	Hy ± SD (mg/g dry plant material)	Hy ± SD (mg/g dry plant material)
**Monoterpene hydrocarbons**			**0.047**	**-**	**-**	**-**
α-Thujene	924	1032	0.023 ± 0.00028	**-**	**-**	**-**
β -Phellandrene	1002	1194	0.024 ± 0.00083	-	-	-
**Oxygenated monoterpenes**			**0.585**	**0.708**	**0.238**	**0.679**
trans -Linalool oxide *	1088	1434	0.010 ± 0.00110	-	-	-
n-Nonanal	1100	1389	0.120 ± 0.00028	0.118 ± 0.00042	-	-
Borneol	1176	1719	0.043 ± 0.00028	0.021 ± 0.00042	-	-
Camphor	1151	1499	0.060 ± 0.00028	0.039 ± 0.00042	-	0.160 ± 0.00045
Pinocarvone	1160	1565	0.055 ± 0.00028	-	-	-
trans-p-Mentha-1(7),8-dien-2-ol	1187	1803	0.212 ± 0.00028	0.220 ± 0.00042	0.220 ± 0.00030	0.288 ± 0.00090
Hexyl 2-methyl butanoate	1233	1425	0.035 ± 0.00028	0.131 ± 0.00126	-	0.197 ± 0.00045
Menthyl acetate	1294	1550	0.050 ± 0.00083	0.179 ± 0.00042	0.018 ± 0.00030	0.034 ± 0.00271
**Sesquiterpene hydrocarbons**			**0.189**	**0.155**	**0.047**	**0.300**
E-Caryophyllene*	1424	1585	0.073 ± 0.00028	0.056 ± 0.00042	0.019 ± 0.00059	0.033 ± 0.00045
δ-Cadinene	1517	1745	0.065 ± 0.00028	-	0.028 ± 0.00177	0.108 ± 0.00362
allo-Aromadendrene	1465	1662	0.042 ± 0.00028	-	-	0.056 ± 0.00045
β-Chamigrene	1478	1724	0.009 ± 0.00028	-	-	-
Germacrene D	1482	1692	-	0.099 ± 0.00042	-	0.103 ± 0.00045
**Oxygenated sesquiterpenes**			**0.088**	**0.398**	**0.285**	**0.194**
Spathulenol	1577	2101	-	-	-	0.056 ± 0.00045
β-Caryophyllene oxide*	1581	1955	0.060 ± 0.00028	0.053 ± 0.00042	0.033 ± 0.00030	0.023 ± 0.00045
γ-Eudesmol	1632	2175	-	-	-	-
α-Muurolol	1645	2181	-	0.052 ± 0.00042	-	-
α-Cadinol	1655	2208	-	0.103 ± 0.00042	-	-
α-Bisabolol	1685	2210	0.015 ± 0.00083	-	0.015 ± 0.00030	0.060 ± 0.00045
α-Bisabolol oxide	1748	2511	-	-	0.009 ± 0.00030	0.023 ± 0.00045
Hexahydrofarnesyl acetone	1839	2113	0.013 ± 0.00028	0.190 ± 0.00168	0.228 ± 0.00059	0.032 ± 0.00045
**Phenolic compounds**			**1.197**	**1.582**	**1.854**	**2.243**
Thymol*	1289	2154	0.230 ± 0.00138	0.397 ± 0.00084	0.103 ± 0.00030	0.189 ± 0.00045
Thymol acetate	1349	-	0.101 ± 0.00028	0.095 ± 0.00042	-	0.110 ± 0.00136
Methyl eugenol	1403	2005	0.834 ± 0.00055	0.980 ± 0.00042	1.713 ± 0.00030	1.891 ± 0.00045
(Z)-Methyl isoeugenol	1451	2070	0.032 ± 0.00028	0.110 ± 0.00252	0.038 ± 0.00030	0.053 ± 0.00045
**Acids, alcohols and esters**			**0.191**	**0.557**	**0.206**	**0.281**
1-Hexadecanol	1874	2371	-	-	0.072 ± 0.00030	-
Hexadecanoic acid	1959	2912	0.126 ± 0.00028	0.264 ± 0.00084	0.067 ± 0.00030	0.085 ± 0.00045
Oleic acid	2133	2998	0.008 ± 0.00028	0.204 ± 0.00042	0.014 ± 0.00030	0.171 ± 0.00045
Octadecanol acetate	2209	-	0.042 ± 0.00028	0.050 ± 0.00042	0.017 ± 0.00059	0.025 ± 0.00045
1-Heptatriacotanol	2309	2309	0.015 ± 0.00028	0.039 ± 0.00042	0.036 ± 0.00030	-
**Hydrocarbons**			**0.279**	**0.380**	**0.114**	**0.461**
Eicosane*	2000	2000	0.042 ± 0.00110	-	0.013 ± 0.00030	0.062 ± 0.00045

Heneicosane*	2100	2100	0.020 ± 0.00028	-	0.009 ± 0.00030	0.025 ± 0.00271
Docosane*	2200	2200	0.032 ± 0.00028	-	0.011 ± 0.00030	0.054 ± 0.00045
Tricosane*	2300	2300	0.017 ± 0.00028	0.036 ± 0.00084	-	-
Tetracosane*	2400	2400	-	0.020 ± 0.00042	0.026 ± 0.00030	0.031 ± 0.00045
Pentacosane*	2500	2500	0.018 ± 0.00028	0.010 ± 0.00042	-	-
Hexacosane*	2600	2600	0.070 ± 0.00028	0.129 ± 0.00042	0.029 ± 0.00059	0.038 ± 0.00136
Heptacosane*	2700	2700	0.087 ± 0.00028	0.135 ± 0.00042	0.009 ± 0.00030	0.047 ± 0.00045
Octacosane*	2800	2800	0.025 ± 0.00028	0.171 ± 0.00042	0.017 ± 0.00059	0.179 ± 0.00045
**Total identification (mg/g)**			**2.576**	**3.670**	**2.744**	**4.158**

Retention indices (RI) were determined relative to a series of *n*-alkanes (C8–C40) on capillary columns VF5-ms (RI^1) [24] and CPWax 52 (RI^2) [25]; Identification method: RI, comparison of RIs with those listed in a homemade library, reported in the literature [24], and/or authentic samples; comparison of mass spectra with those in mass spectral libraries NIST02 and Wiley 9; * co-injection with reference compounds; SD, standard deviation.

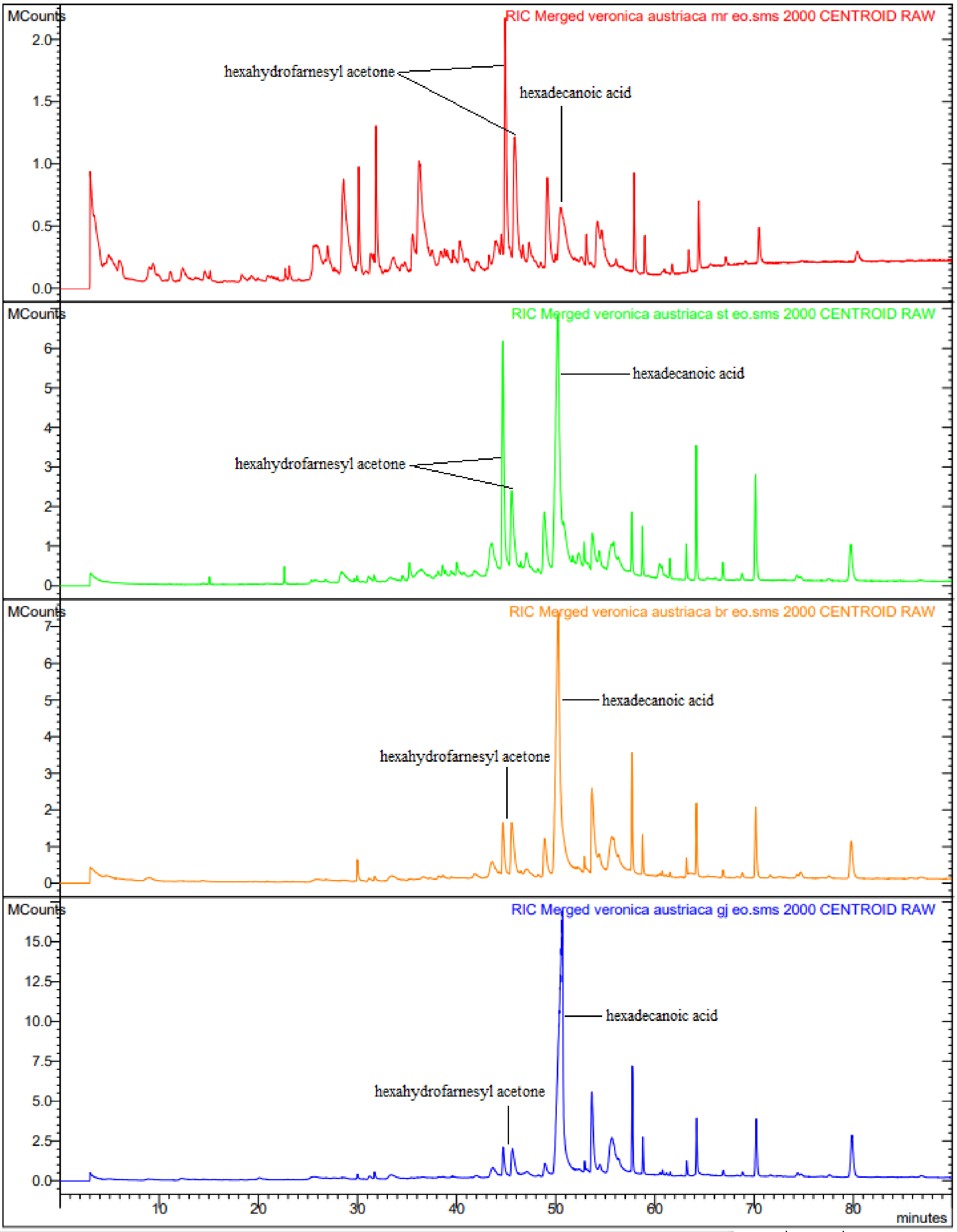

Figure S1. RIC chromatograms of the four samples of EOs of *V. jacquinii* with marked major compounds

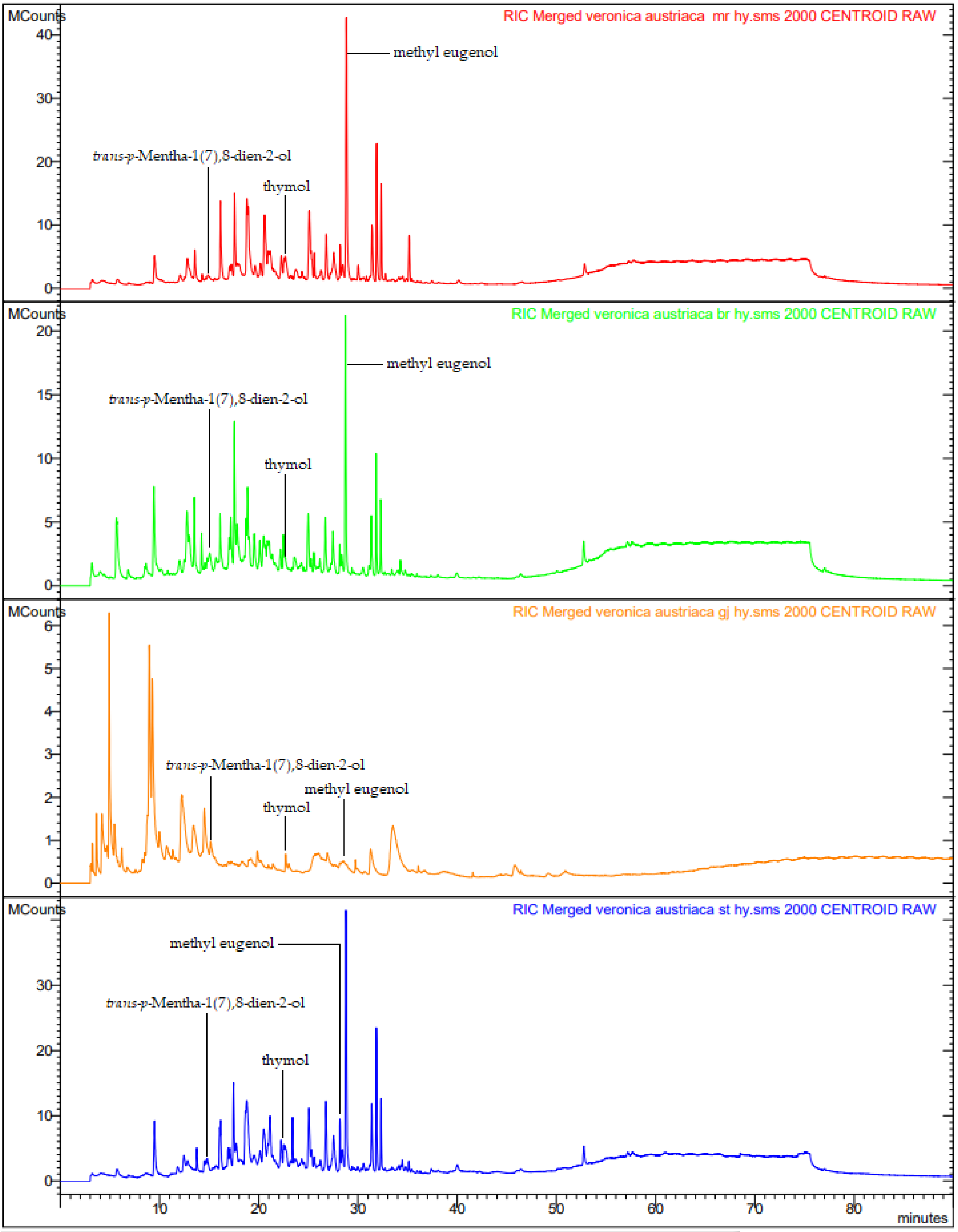

Figure S2. RIC chromatograms of the four samples of hydrosols of *V. jacquinii* with marked major compounds
